# The effect of proximal anastomosis on the expansion rate of a dilated ascending aorta in coronary artery bypass surgery: a prospective study

**DOI:** 10.5830/CVJA-2016-071

**Published:** 2017

**Authors:** Ahmet Yavuz Balcı, Unsal Vural, MD Fatih Özdemir, Mehmet Kızılay, Mutlu Şenocak, Ilyas Kayacıoğlu, Ibrahim Yekeler, Rezan Aksoy, Seçkin Satılmış,, Huseyin Şaşkın

**Affiliations:** Department of Cardiovascular Surgery, Dr Siyami Ersek Cardiovascular Surgery and Thoracic Hospital, Istanbul, Turkey; Department of Cardiovascular Surgery, Dr Siyami Ersek Cardiovascular Surgery and Thoracic Hospital, Istanbul, Turkey; Department of Cardiovascular Surgery, Dr Siyami Ersek Cardiovascular Surgery and Thoracic Hospital, Istanbul, Turkey; Department of Cardiovascular Surgery, Dr Siyami Ersek Cardiovascular Surgery and Thoracic Hospital, Istanbul, Turkey; Department of Cardiovascular Surgery, Dr Siyami Ersek Cardiovascular Surgery and Thoracic Hospital, Istanbul, Turkey; Department of Cardiovascular Surgery, Dr Siyami Ersek Cardiovascular Surgery and Thoracic Hospital, Istanbul, Turkey; Department of Cardiovascular Surgery, Dr Siyami Ersek Cardiovascular Surgery and Thoracic Hospital, Istanbul, Turkey; Department of Cardiovascular Surgery, Koşuyolu Training and Research Hospital, Istanbul, Turkey; Department of Cardiology, Acibadem University, Istanbul, Turkey; Department of Cardiovascular Surgery, Derince Training and Research Hospital, Istanbul, Turkey

**Keywords:** coronary artery bypass grafting, aortic dilatation, proximal anastomosis

## Abstract

**Background:**

This study was designed to determine the short- and long-term effects of proximal aortic anastomosis, performed during isolated coronary artery bypass grafting (CABG) in patients with dilatation of the ascending aorta who did not require surgical intervention.

**Methods:**

The study was performed on 192 (38 female and 160 male patients; mean age, 62.1 ± 9.2 years; range, 42–80 years) patients with dilatation of the ascending aorta who underwent CABG surgery between 1 June 2006 and 31 May 2014. In group 1 (n = 114), the saphenous vein and left internal mammarian artery grafts were used, and proximal anastomosis was performed on the ascending aorta. In group 2 (n = 78), left and right internal mammarian artery grafts were used, and proximal aortic anastomosis was not performed. Pre-operatively and in the first and third years postoperatively, the ascending aortic diameter was measured and recorded using transthoracic echocardiography at four different regions (annulus, sinus of Valsalva, sinotubular junction and tubular aorta).

**Results:**

A statistically significant difference was found between the groups for the number of grafts used and the duration of aortic cross-clamping and cardiopulmonary bypass. No significant intergroup difference was seen for the mean diameter of the ascending aorta (p > 0.05). Annual changes in the aortic diameter were found to be extremely significantly different in both groups (p = 0.0001). Mean values of the aortic diameter at the level of the sinotubular junction and tubular ascending aorta, mean aortic diameters (p = 0.002 and p = 0.0001, respectively), annual increase in diameter (p = 0.0001 and p = 0.0001, respectively), and mean annual difference in diameter (p = 0.0001 and p = 0.0001, respectively) at one and three years postoperatively were statistically significantly different between the groups.

**Conclusion:**

In patients with ascending aortic dilatation who did not require surgical intervention and who had proximal anastomosis of the ascending aorta and underwent only CABG, we detected statistically significant increases in the diameter of the sinotubular junction and tubular aorta up to three years postoperatively.

## Background

Aortic dilatation is a clinical entity with many aetiological factors, which can be seen singly or in association with other cardiac pathologies. Generally, anuloaortic ectasia, Marfan syndrome, atherosclerotic aortic degeneration, aortic dissection and bicuspid aortic valve disease accompany aortic dilatation.^1^

Despite novel approaches in surgical and endovascular treatment procedures for aneurysms, the same degree of advancement has not been achieved with regard to the molecular and cellular mechanisms that trigger its pathogenesis and progression. Genetic factors and inflammatory responses are recognised as important aspects in the development of aneurysms. Fedak et al. reported the fundamental role of matrix metalloproteinases (MMP) in the structural integrity of the aorta.^2^

In some studies, loss of elastin and collagen of the aortic wall has been demonstrated to induce the development of aneurysms. Loss of elastin and collagen is caused by an increase in the activity of matrix proteinases (elastase and collagenase) or a decrease in the activity of anti-proteases [mellaproteinase tissue inhibitor (TIMP)] and alpha-1-antitrypsin.^2^ Increase in the levels of intercellular adhesion molecules induces migration of macrophages to this region, with the resultant increase in the production of MMP2 and MMP9, fragmentation of elastin and triggering of aneurysm formation. Macrophages degrade elastin by activating tumour necrosis factor and interleukin-1. The production of elastin and collagen may also be impaired due to genetic causes, as seen in Marfan syndrome and Ehlers Danlos type 4.^2^

Knowing the aetiological factors causing aneurysms may contribute to slowing down the pathogenic process and determination of a treatment modality. Aortic valve pathologies, hypertension, smoking, alcoholic beverages, diabetes mellitus, cross-clamping, cannulation site, aortic suture lines, and proximal anastomosis of coronary artery grafts have been held responsible for the development of aortic aneurysms. Formulae suggested to estimate the growth rate of aortic aneurysms demonstrate differences based on aetiological, regional and geographic conditions.^3^

This study was designed to determine the short- and longterm effects of proximal aortic anastomosis performed during isolated coronary artery bypass grafting (CABG) in patients with dilatation of the ascending aorta who did not require surgical intervention.

## Methods

The study, to be performed on patients with dilatation of the ascending aorta who would undergo CABG surgery in the clinics of Dr Siyami Ersek Thoracic and Cardiovascular Surgery Training and Research Hospital between 1 June 2006 and 31 May 2014, was initiated after approval of the local ethics committee was obtained. The objective of the study was explained to all patients and their written approval was obtained. The study was completed with 192 patients (38 female and 160 male; mean age 62.1 ± 9.2 years; range 42–80 years) who had to undergo isolated CABG surgery.

Patients with a diagnosis of connective tissue disease, those who had undergone additional cardiac surgery, re-operation, cases with aneurysms at various regions of the aorta or peripheral arteries, individuals with extremely calcified aortae and congenital or acquired aortic valve pathologies, and patients lost to postoperative follow up were excluded from the study. Patients who had cardiopulmonary bypass (CPB) and isolated CABG and those whose ascending aortic diameter was 40–45 mm (mean: 42.1 ± 1.8 mm) at its widest region, determined using transthoracic echocardiography (TTE), were included in the study.

The patients were divided into two groups. In group 1 (n = 114, 59.4%), saphenous vein and left internal mammarian artery (LIMA) grafts were used, and proximal anastomosis was performed on the ascending aorta. In group 2 (n = 78, 40.6%), LIMA and right internal mammarian artery (RIMA) grafts were used, and proximal aortic anastomosis was not performed.

Clinical and demographic data of the patients related to age and gender, left ventricular ejection fraction (LVEF), hypertension (HT), diabetes mellitus (DM), chronic obstructive pulmonary disease (COPD), chronic renal failure (CRF), previous myocardial infarction (MI), hyperlipidaemia, peripheral artery disease (PAD), stroke, smoking status and alcohol use were recorded. Pre-operatively and in the first and third years postoperatively, the ascending aorta was measured and recorded using TTE diameters at four different regions (annulus, sinus of Valsalva, sinotubular junction and tubular aorta). Postoperative monitoring of the patients was achieved via communication with patients by telephone.

Under routine intra-operative anaesthesia, a median sternotomy was performed on all patients. The bypass grafts (LIMA, RIMA, saphenous vein) were prepared. Following heparinisation (3 mg/kg IV), an arterial cannula was inserted into the ascending aorta and a two-stage cannula was implanted into the right atrium. Using a roller pump and membrane oxygenator, we proceeded with CPB.

During CPB, activated coagulation time was maintained over 400 seconds. Moderate levels of systemic hypothermia (28–30°C) were used. Pump flow rate and perfusion pressure were held at 2.2–2.4 l/min/m^2^ and 50–85 mmHg, respectively. Following crossclamping of the aorta, cold blood cardioplegia was performed in the antegrade direction to achieve cardiac arrest. After completion of the distal anastomosis, cardioplegic solution was delivered through the saphenous vein graft and myocardial protection was maintained. After placement of the side clamps, proximal anastomosis was performed on a beating heart.

The patients were extubated in the intensive care unit within three to six hours of the operation. As criteria for extubation, the patient had to be wide awake, haemodynamically stable, and the amount of hourly drainage had to have dropped to acceptable amounts. During the postoperative period, patients who did not develop major complications were followed up in the ward. All patients were discharged after an average of seven to nine days.

As a control, TTE was performed at one and four weeks postoperatively and no pathological evidence was found. TTE was repeated at one and three years postoperatively. The TTE procedure was performed by a cardiologist blinded to the grouping of patients. Before the procedure, the patients were informed about the procedure and their approvals were obtained.

Measurements were made at four different regions of the ascending aorta. During TTE, ventricular and valvular dysfunction (if any) were also determined. Left ventricular end-diastolic (LVEDD) and end-systolic diameters (LVESD), LVEF, and systolic and diastolic volumes were also determined. In our study we chose TTE rather than CT angiography as TTE provides information on ventricular and valvular function and evaluates the aortic annulus and sinotubular junction more effectively, in addition to its lower cost and non-toxicity.

## Statistical analysis

For statistical evaluations, the SPSS statistical program (SPSS for Windows, version 11.0, SPSS Inc, Chicago) was used. If all measured data demonstrated a normal distribution, they were expressed as mean ± standard deviation; if not, they were indicated as median (minimum–maximum) values. Numerical data were presented as percentages (%).

For data obtained with measurements, normality of distribution was evaluated using histograms or the Kolmogorov– Smirnov test and their homogeneity were assessed with Levene’s test for equality of variance. For data with normal and homogenous distribution, intergroup difference was evaluated using the Student’s t-test, while data with non-normal and non-homogenous distributions were evaluated using the Mann– Whitney U-test.

Intergroup differences among the numerical data were evaluated with parametric or non-parametric Pearson’s chi-squared and Fisher’s exact test, based on the parametric or non-parametric distribution of data, respectively. In comparisons of mean values of dependent groups, Friedman’s S-test was used. Data with a p-value < 0.05 was accepted as statistically significant.

## Results

Demographic characteristics and clinical features of the patients with aortic dilatation who underwent CPB and isolated CABG surgery are summarised in Table 1. No statistically significant differences were found between the groups for demographic and clinical characteristics (p > 0.05).

**Table 1:** Demographic and clinical characteristics of the patients

**	*Group 1 (n = 114) Presence of proximal anastomosis*	*Group 2 (n = 78) Absence of proximal anastomosis*	*p-value*
Mean age (years) (median, range)	63.0 ± 9.2 (63, 42–80)	61.0 ± 9.3 (61, 42–80)	0.12**
Male, n (%)	93 (60.4)	61 (39.6)	0.56*
Female, n (%)	21 (55.3)	17 (44.7)	0.45*
Ejection fraction (%) (median, range)	53.5 ± 10.2 (55, 30–70)	53.7 ± 10.5(55, 30–70)	0.91**
Hypertension, n (%)	28 (24.6)	27 (34.6)	0.13*
Diabetes mellitus, n (%)	43 (37.7)	37 (47.4)	0.18*
Smoking status, n (%)	34 (29.8)	19 (24.4)	0.41*
Hyperlipidemia, n (%)	44 (38.6)	34 (43.6)	0.49*
COPD, n (%)	11 (9.6)	5 (6.4)	0.43*
Pre-operative CRF, n (%)	6 (3.3)	2 (2.6)	0.48***
Peripheral artery disease, n (%)	8 (7.0)	3 (3.8)	0.53***
History of stroke, n (%)	7 (6.1)	2 (2.6)	0.32***
Previous myocardial infarction, n (%)	30 (26.3)	23 (29.5)	0.63*
Alcohol use, n (%)	15 (13.2)	8 (8.3)	0.54*

*Pearson’s chi-squared test; **Mann–Whitney U-test; ***Fisher’s exact test. COPD = chronic obstructive pulmonary disease; CRF = chronic renal failure.

The mean number of grafts used in the CABG operation was calculated for group 1 [3.6 ± 0.9 (median: 4; range: 1–6)] and group 2 [1.5 ± 0.5; (median: 1; range: 1–2)]. A statistically significant intergroup difference was found (p = 0.0001).

Mean aortic cross-clamping durations were 54.9 ± 11.5 min (median: 65.5, range: 23–76) in group 1 and 26.7 ± 6.4 min (median: 25, range: 18–35) in group 2. A statistically significant intergroup difference was found between the groups (p = 0.0001).

Mean CPB durations were 82.8 ± 14.3 min (median: 84, range: 39–109) in group 1 and 46.7 ± 6.3 min (median: 46, range: 35–58) in group 2 (p = 0.0001). Total mean intubation times were 5.6 ± 1.9 hours (median: 5, range: 3–14) in group 1 and 5.5 ± 1.2 hours (median: 5, range: 4–12) in group 2. Mean duration of stay in the postoperative intensive care unit was 29.3 ± 17.1 hours (median: 21, range: 17–88) in group 1 and 29.7 ± 15.3 hours (median: 22, range: 17–71) in group 2, with no significant intergroup differences (p = 0.53).

Mean hospital stay of the patients was 6.1 ± 1.6 days (median: 5, range: 5–12) in group 1 and 5.8 ± 1.1 days (median: 5, range: 5–9) in group 2 with no significant intergroup differences (p = 0.83). In group 1, 48 (42.2%) and in group 2, 35 (44.9%) patients required transfusion of blood and blood products during the postoperative period, with no statistically significant difference between the groups (p = 0.70). The mean total amount of postoperative drainage was 348 ± 169 ml (median: 300, range: 150–1100) in group 1 and 335 ± 125 ml (median: 300, range: 150–700) in group 2, with no statistically significant intergroup difference (p = 0.93).

**Fig. 1.**
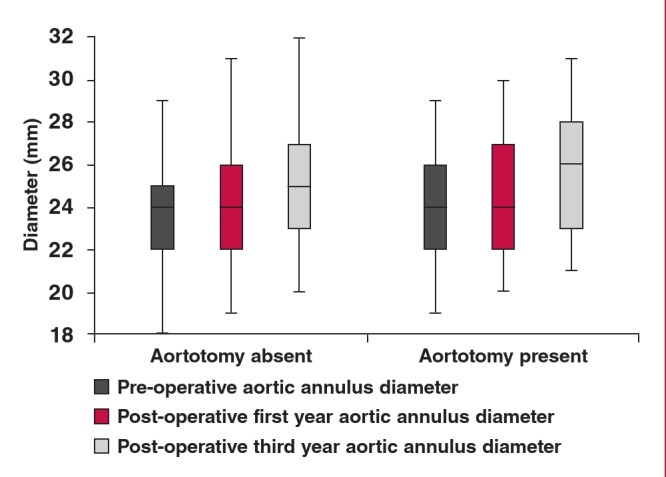
Measurements of the aortic annulus.

Changes in the aortic root and ascending aortic diameter over three years and analysis of this change are shown in Table 2. No statistically significant intergroup difference was seen at the level of the aortic annulus (Fig. 1) (p = 0.22, p = 0.25 and p = 0.13, respectively). Annual changes in aortic diameter were found to be extremely significantly different in both groups (p = 0.0001). However, when mean differences in diameters were analysed by year, no statistically significant intergroup difference was detected (p = 0.21 and p = 0.37, respectively).

**Table 2:** Comparison of transthoracic echocardiography measurements and annual differences

**	*Group 1 (n = 114) Presence of proximal anastomosis*	*Group 2 (n = 78) Absence of proximal anastomosis*	*p-value*
Pre-operative annulus diameter (mm) (median, range)	24.2 ± 2.8 (24, 19–29)	23.7 ± 3.1 (24, 18–31)	0.22^a^
1st year annulus diameter (mm) (median, range)	24.6 ± 2.7 (25, 20–30)	24.2 ± 2.9 (24, 19–31)	0.25^a^
3rd year annulus diameter (mm) (median, range)	25.6 ± 2.8 (26, 21–31)	25.0 ± 2.8 (25, 20–32)	0.13^a^
p-value	0.0001^b^	0.0001b^b^	
1st year annulus diameter difference (mm) (median, range)	0.37 ± 0.50 (0, 0–2)	0.46 ± 0.53 (0, 0–2)	0.21^a^
3rd year annulus diameter difference (mm) (median, range)	1.37 ± 0.55 (1, 1–3)	1.27 ± 0.70 (1, 0–3)	0.37^a^
p-value	0.0001^b^	0.0001^b^	
Pre-operative sinus of Valsalva diameter (mm) (median, range)	37.2 ± 1.6 (37, 34–40)	37.1 ± 1.6 (37, 34–41)	0.53^a^
1st year sinus valsalva diameter (mm) (median, range)	38.0 ± 1.7 (38, 35–41)	37.8 ± 1.6 (38, 35–42)	0.37^a^
3rd year sinus of Valsalva diameter (mm) (median, range)	38.8 ± 1.6 (39, 36–42)	38.5 ± 1.7 (39, 35–42)	0.30^a^
p-value	0.0001^b^	0.0001^b^	
1st year sinus of Valsalva diameter difference (mm) (median, range)	0.79 ± 0.43 (1, 0–2)	0.76 ± 0.49 (1, 0–2)	0.57^a^
3rd year sinus of Valsalva diameter difference (mm) (median, range)	1.63 ± 0.49 (2, 1–2)	1.45 ± 0.53 (1, 1–3)	0.01^a^
p-value	0.0001^b^	0.0001^b^	
Pre-operative STJ diameter (mm) (median, range)	39.1 ± 1.1 (39, 33–41)	38.9 ± 1.6 (39, 36–42)	0.08^a^
1st year STJ diameter (mm) (median, range)	40.3 ± 1.1 (40, 37–42)	39.7 ± 1.6 (39.5, 36–43)	0.002^a^
3rd year STJ diameter (mm) (median, range)	41.4 ± 1.2 (41.5, 38–44)	40.3±1.7 (40, 37–45)	0.0001^a^
p-value	0.0001^b^	0.0001^b^	
1st year STJ diameter difference (mm) (median, range)	0.99 ± 0.36 (1, 0–2)	0.76 ± 0.49 (1, 0–2)	0.0001^a^
3rd year STJ diameter difference (mm) (median, range)	2.21 ± 0.66 (2, 0–3)	1.35 ± 0.88 (1, 0–3)	0.0001^a^
p-value	0.0001b	0.0001b	
Pre-operative ascending aorta diameter (mm) (median, range)	41.5 ± 1.3 (41, 40–45)	41.6 ± 1.6 (41, 40–45)	0.79^a^
1st year ascending aorta diameter (mm) (median, range)	43.0 ± 1.5 (43, 40–48)	42.1 ± 1.5 (42, 40–45)	0.0001^a^
3rd year ascending aorta diameter (mm) (median, range)	44.3 ± 1.6 (44, 42–49)	42.9 ± 1.6 (43, 40–46)	0.0001^a^
p-value	0.0001^b^	0.0001^b^	
1st year ascending aorta diameter difference (mm) (median, range)	1.51 ± 0.76 (2, 0–3)	0.58 ± 0.57 (1, 0–2)	0.0001^a^
3rd year ascending aorta diameter difference (mm) (median, range)	2.77 ± 0.95 (3, 1–5)	1.32 ± 0.57 (1, 0–3)	0.0001a
p-value	0.0001^b^	0.0001^b^	

^a^Mann–Whitney U-test; bFriedman S-test; STJ = sinotubular junction.

**Fig. 2.**
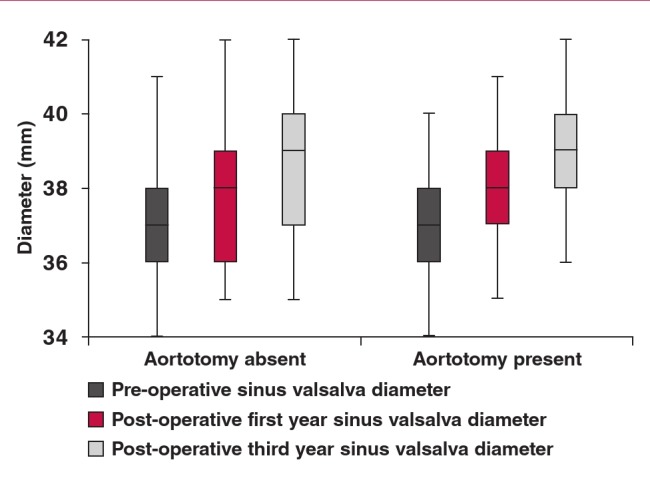
Measurements of the sinus of Valsalva.

Mean values of aortic diameter at the level of the sinus of Valsalva were not significantly different between the groups (Fig. 2) (p = 0.53, p = 0.37 and p = 0.30, respectively). However, annual increases in diameter were found to be extremely significantly different in both groups (p = 0.0001 and p = 0.0001, respectively). The mean difference in diameter one year postoperatively was not statistically significantly different between the groups (p = 0.57), while at three years, the intergroup difference was statistically significantly different (p = 0.01).

**Fig. 3.**
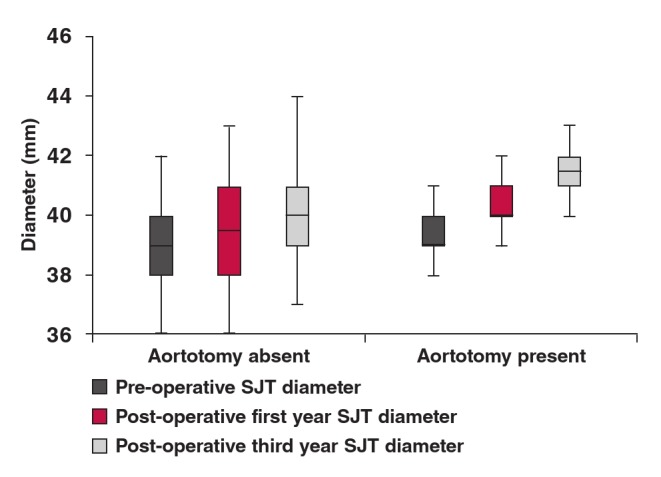
Measurements of the sinotubular junction.

Pre-operatively at the level of the sinotubular junction, the mean aortic diameters were not significantly different between the groups (Fig. 3) (p = 0.08), while the mean aortic diameters at one and three years postoperatively were statistically significantly different between the groups (p = 0.002 and p = 0.0001, respectively). Annual increase in diameter was extremely significantly different between the groups (p = 0.0001 and p = 0.0001, respectively). Mean annual difference in diameter was found to be extremely significantly different between the groups (p = 0.0001 and p = 0.0001, respectively).

**Fig. 4.**
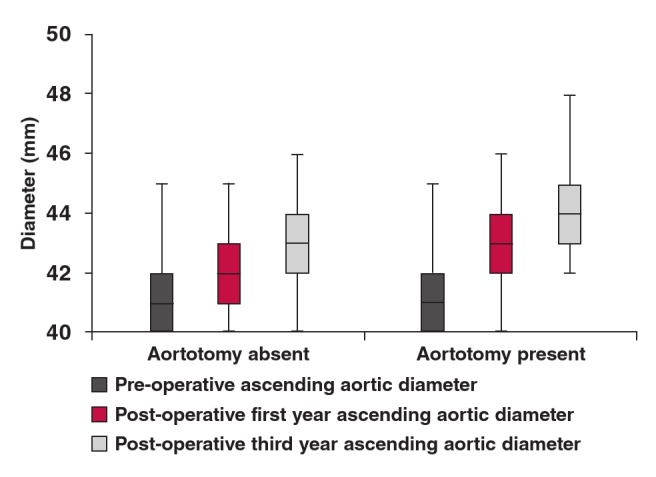
Measurements of the ascending aorta.

Pre-operatively, mean aortic diameters measured at the level of the tubular ascending aorta were not significantly different between the groups (Fig. 4) (p = 0.79). However, mean aortic diameters measured one and three years postoperatively were extremely significantly different between the groups (p = 0.0001 and p = 0.0001, respectively). The increase in diameter of the ascending aorta was extremely significantly different in both groups (p = 0.0001 and p = 0.0001, respectively). Intergroup differences in mean value of the diameter of the ascending aorta at one and three years postoperatively were extremely significantly different (p = 0.0001 and p = 0.0001, respectively).

## Discussion

Since proximal anastomosis of the coronary artery graft to the ascending aorta requires aortotomy, aortic integrity is disrupted during this procedure. Besides, coronary artery grafts in their new position will increase the haemodynamic workload of the ascending aorta and, theoretically, they can be seen as a cause of aortic dilatation. However, no clinical or experimental study in the literature has held coronary artery grafts responsible for aortic dilatation.

We could also not find any study in the literature evaluating the relationship between mid- and long-term increase in aortic diameter and proximal anastomosis performed on the ascending aorta in patients with aortic dilatation who did not require surgical intervention but underwent CPB and isolated CABG. We searched for the words ‘aortic dilatation’, ‘proximal anastomosis’ and ‘coronary artery bypass grafting’ in the English literature of PubMed, but could not find any article related to this topic.

No study could be found in the literature referring to aortic dilatation caused by aortic side clamping. Therefore no assessment was done in our study on the effects of aortic side clamping versus aortotomy. New studies are needed to determine whether there is a difference. We rarely undertake proximal anastomosis to the aorta using the aortic cross-clamp, we prefer the aortic side-clamp.

In this study, we tried to determine whether proximal anastomosis performed on the ascending aorta in patients who had undergone CPB and isolated CABG surgery had any effect on the mean increase in aortic diameter during the early and mid-term postoperative period. Arteriotomy on dilated aortae for proximal anastomosis of saphenous vein grafts to the aorta leads to disruption of the elastic structure and connective tissue integrity of the aorta. How this affects the dilatation of the aorta is unknown. This study aimed at answering that question.

In our study, we found that annual increases in all segments of the aorta were statistically significantly different in both groups. In patients who had undergone proximal anastomosis of the ascending aorta, increases in the diameter of the sinotubular junction and ascending aorta at one and three years postoperatively were significantly greater than in patients who had not undergone a proximal anastomosis. We believe that this strengthens our hypothesis, which asserted that surgical manipulation on a dilated ascending aorta increases the speed of aortic expansion.

An aortic diameter exceeding normal limits, based on the patient’s age and body surface area, is termed aortic dilatation, and if it increases more than 50% of normal, it is termed aortic aneurysm.^4^ Aneurysm of the ascending aorta is a frequently seen clinical condition. Haemodynamic force, degradation of the configured extracellular matrix, familial predisposition and transmural inflammation have been demonstrated as aetiological factors for this disease.^5^

Aortic wall strain acts in direct proportion with aortic diameter and pressure, while it is in inverse proportion with aortic wall thickness, in compliance with Laplace’s law [wall strain = pressure × radius (r)/2 × wall thickness (h)].^6^ The wall of the enlarged aorta has a risk of rupture. Recent studies have reported that aneurysms with a diameter less than 50 mm have a 2% annual risk of rupture.^6^ However, as reported in various studies, for aneurysms with a diameter of 60 mm, annual risk of rupture and mortality increases up to 11.8 and 6.9%, respectively. The probability of their combined risk has been reported at 14.1%.^7^

In patients with aneurysms of the ascending aorta who will undergo valvular surgery, dilatations of less than 50 cm, unexplained dilatations of ≥ 55 mm, patients with Marfan syndrome and bicuspid aortic dilatations of ≥ 50 mm, and for smaller dilatations with an annual growth rate of 10 mm, surgery is recommended.^8^ In our patients, the aortic diameter was 40–45 mm (mean 41.5 ± 1.4 mm) without any connective tissue disease as aetiological factor, so we did not consider any indication for surgery in our patients.

Postoperative dilatation rate is important because of the risks of dilatation and rupture. Expansion rate of the ascending aorta with a diameter of 40 mm following aortic valve surgery has been reported as 0.5–2.4 mm/year (mean 0.45 mm/year).^9^,^10^ However Andrus et al. found an expansion rate after aortic valve replacement (AVR) of –0.1 mm/year. This suggests that AVR changes the natural course of aortic dilatation.^11^

Keane et al. reported that ascending aortae of patients with bicuspid valves are more frequently prone to dilatation.^9^ In their series of 14 cases, Yasuda et al. followed their patients for 10 years and reported an annual aortic expansion rate of 0.08 mm/year. In their studies using CT angiography and echocardiography for evaluation, they indicated that an increase in the aortic diameter of 0.2–0.3 mm within 10 years was not statistically significant. In our study, postoperative (group 2) dilatation in cases with tricuspid aortic valves had an annual dilatation rate of 1 mm/year (Table 2). We believe that this dilatation rate was related to risk factors independent of surgery and valvular pathology.

Natsuaki et al. reported that patients who underwent mechanical valve implantation carried a higher risk of aortic dissection and rupture when compared with those who had received biological valves.^12^ This contradicts the belief that biological valves leave behind greater residual gradient and undergo faster degeneration in sepsis and infection as endocarditis. In our patients during the three years of follow up, we did not observe aortic dissection and rapid development of aneurysmatic dilatation (Fig. 1). However, cases with post-CABG dissection have occasionally been reported in the literature.^13^

Mortality rates in cases of surgery of the ascending aorta have been reported to range between 1.7 and 17.1% and in re-operated cases, between 6 and 32%.^14^,^15^ The surgical procedures used and aetiological factors are known determinants of mortality.^13^ In their series, Atik et al. detected aortic dilatations in three (17%) patients following coronary artery surgery.^14^ Songur et al. reported aortic dilatation in 50 cases within nine years of cardiac surgery; eleven (22%) of these cases with aortic dilatation developed after CABG. In these cases, average diameter of the ascending aorta after the first and second operation was indicated as 4.1 and 5.5 cm, respectively.^16^ Aortic manipulation (proximal anastomosis line, cannulation, suture lines, crossclamping injury) and aortic valve pathologies have been held responsible for the development of these dilatations. No case– control studies where aortic valves were evaluated following proximal anastomosis have been performed.

In our cases, we detected a dilatation rate of 3.04 mm over three years (7.1%) at the level of the tubular segment of the ascending aorta. In the patients who underwent proximal anastomosis, aortic dilatation was more severe (median 3.7 mm per three years; 8.7%) but the intergroup difference was not statistically significantly different (p = 0.059). In the first postoperative year, the intergroup difference was significantly different at the level of the tubular aorta (p = 0.02). Dilatation of all segments of the ascending aorta over time was statistically significantly different in both groups (p = 0.001; Table 2). Intergroup difference in the tubular ascending aorta in the first year could have been related to the proximal anastomosis. However when the magnitude of standard deviation and width of confidence intervals are taken into consideration, a confounding effect of aetiological factors (connective tissue disease) should not be overlooked (Fig. 1).

The causative effects of risk factors such as diabetes mellitus, hypercholesterolaemia, age, hypertension, smoking and alcohol abuse on atherosclerosis are well recognised.^17^ Narrowings or occlusions occur in the vasa vasorum of the atherosclerotic aorta, which result in an increase in the levels of elastase enzyme, a decrease in the levels of anti-protease enzyme and degradation of the elastin. Consequently, aneurysmatic dilatations develop on the weakened vascular wall.^18^,^19^ Matsuyama et al. detected a higher number of patients with PAD, TIA, stroke, current and past smoking history and COPD among those who had developed aortic dilatation following AVR.^20^ However, they reported a lower incidence of aortic dilatation in patients who used beta-blockers and those with calcified aortae.^21^

In our patients, the presence of COPD, smoking, stroke and beta-blocker use did not differ between the groups (p > 0.05) (Table 3). However, during the three years of follow up, only a minimal contribution of COPD to aortic dilatation at the level of the sinus of Valsalva was detected (Table 3). In patients with diabetes, the expansion velocity was observed to be 0.4 mm/year, being most marked at the level of the sinotubular junction.

**Table 3:** The effect of pre-operative risk factors on changes in aortic diameter at three years of follow up

*Difference in diameter of the ascending aorta (mm/3 years)*			
**	*Aortic ring*	*Sinus of Valsalva*	*Sinotubular junction*	*Tubular aorta*
Hypertension				
Present	1.27 ± 1.1	1.5 ± 0.37	1.5 ± 1.2	6.7 ± 1.9
Absent	1.1 ± 1.3	1.6 ± 0.38	1.1 ± 1.1	2.1 ± 1.7
p-value^a^	0.488	0.501	0.01	0.001
Smoking				
Present	1.1 ± 1.2	1.6 ± 0.34	0.8 ± 1.0	3.7 ± 3.2
Absent	1.2 ± 1.3	1.5 ± 0.45	1.1 ± 1.1	3.3 ± 2.5
p-value^a^	0.756	0.163	0.004	0.793
Hypercholesterolaemia				
Present	1.1 ± 1.3	1.6 ± 0.4	1.4 ± 1.2	3.0 ± 2.6
Absent	1.2 ± 1.2	1.6 ± 0.41	1.1 ± 1.0	3.7 ± 2.7
p-value^a^	0.852	0.401	0.222	0.97
Alcohol abuse								
Present	0.75 ± 1.0	1.8 ± 0.3	1.1 ± 1.3	3.9 ± 3.0
Absent	1.2 ± 1.2	1.6 ± 0.4	1.2 ± 1.1	3.4 ± 2.7
p-value^a^	0.282	0.01186	0.745	0.540
Diabetes mellitus				
Present	1.1 ± 1.2	1.7 ± 0.3	1.2 ± 1.1	3.3 ± 4.2
Absent	1.7 ± 1.7	1.6 ± 0.4	0.4±0.7	3.4 ± 2.6
p-value^a^	0.095	0.475	0.01	0.195
Chronic obstructive pulmonary disease				
Present	1.5 ± 0.7	1.6 ± 0.4	1.4 ± 1.0	3.1 ± 1.9
Absent	1.1 ± 1.3	1.4 ± 0.3	1.2 ± 1.1	3.4 ± 2.7
p-value^a^	0.295	0.032	0.233	0.755
Gender				
Female	1.3 ± 1.0	1.5 ± 1.2	1.3 ± 1.2	3.6 ± 2.0
Male	1.4 ± 0.9	1.4 ± 1.1	1.2 ± 1.1	3.5 ± 2.2
p-value^a^	0.752	0.252	0.920	0.178
Beta-blocker (+)				
Present	1.2 ± 0.0	1.4 ± 1.2	1.3 ± 1.2	3.0 ± 1.9
Absent	1.3 ± 0.0	1.5 ± 1.1	1.2 ± 1.3	3.9 ± 2.1
p-value^a^	0.190	0.082	0.178	0.098

^a^Mann–Whitney U-test.

Hypertension is known to be an independent risk factor for aortic dilatation. In various studies, annual increases in the diameter of the ascending aorta have been reported at 1.25 mm in normotensive and 2.8 mm in hypertensive patients.^21^ However, since these patients are continually on drug therapy, it is difficult to investigate the effects of uncontrolled hypertension on annual expansion rates. In the presence of controlled hypertension and diabetes, dilatation of the sinotubular junction and tubular segments of the aorta was more frequently observed. In tractable hypertension, annual dilatation rate at the level of the tubular aorta was found to be 2.2 mm.^21^

In our study, age demonstrated a significant correlation with dilatation rates in both groups for all segments of the aorta, excluding the aortic ring. Other risk factors made minimal contributions to dilatation rates, with no statistically significant differences (p > 0.05) (Table 3).

Various formulae have been developed to predict the expansion rate of aortic aneurysms, but no correlation between the determined and estimated size of the aneurysm could be demonstrated.^22^ Bonser et al. followed up the natural course of aneurysms in their series and reported an annual aortic expansion rate of 3.3 mm for 44-mm-dilated aneurysms associated with thrombi, but without any evidence of stroke and TIA. The annual expansion rate was 1.9 mm without the concurrent presence of thrombi. In this series, decreased growth rates were reported for larger aneurysms.^22^

Similarly, segments proximal and distal to the aneuryms were tracked in patients who had previously undergone aortic surgery, and a decrease in expansion rate of the aneurysms to 1.18–1.59 mm/year was reported.^19^ In our cases, annual expansion rates differed in various aortic regions, while the tubular (mid-) segment of the ascending aorta was the most dilated portion. Irrespective of aetiological factors, the mean expansion rate of this segment was 1.2 ± 0.9 mm/year, which was similar to the dilatation rate following AVR.

On TTE, measurements of the aortic diameter may demonstrate individual differences. CT angiography has a higher sensitivity and specificity for the ascending aorta. However, in multi-slice sections, it is difficult to evaluate the sinotubular junction and annulus. In addition to the higher cost of CT angiography, the contrast material used carries risks of anaphylaxis and renal toxicity. Therefore, we deemed it appropriate to analyse our cases using TTE, which allows evaluation of ventricular and valvular function.

## Limitations

Pre-operatively, the aortic diameters of our patients ranged between 40 and 45 mm. Since it is known that the expansion rate decreases in proportion to the increase in aortic diameter, we believe that comparative regression analysis between groups of aortae with varying diameters would reveal a correlation. Although our follow-up period was only three years, we obtained values close to those cited in the literature. However, we are of the opinion that in long-term follow-up studies (five to 10 years), more significant and precise results would be obtained.

Genetic investigations were not done in our cases or in other studies on this subject. When undertaking genetic investigations, if patients with connective tissue disorders are grouped separately, then the number of extreme values would decrease and these groups would demonstrate a more homogenous distribution, with similar effects on the outcomes.

## Conclusion

In our three-year follow-up study on patients with ascending aortic dilatation that did not require surgical intervention, who underwent proximal anastomosis of the ascending aorta and only CABG surgery, we detected significant increases in diameters of the sinotubular junction and tubular aorta. Since our study population was homogenous as far as the demographic and clinical characteristics were concerned, and their hypertension was under control, we believe that this statistically significant postoperative increase in expansion rate of dilated aorta was induced by the surgical interventions performed on the aorta. However, since clinical progression of these cases is unpredictable, periodic echocardiographic or CT angiographic monitoring of these patients is required, with regard to the development of aortic dissection and aneurysm.
